# Long-Term Outcomes of Living-Related Kidney Donors Over 26 Years: A Study of 154 Cases From the Ibn Sina University Hospital Center in Rabat, Morocco

**DOI:** 10.7759/cureus.110896

**Published:** 2026-06-15

**Authors:** Salima Serroukh, Qods Yacoubi, Nadia Sakout, Amal Zniber, Loubna Benamar, Naima Ouzeddoun

**Affiliations:** 1 Department of Nephrology, Dialysis and Kidney Transplantation, Ibn Sina University Hospital Center, Rabat, MAR; 2 Faculty of Medicine and Pharmacy, Mohammed V University, Rabat, MAR

**Keywords:** chronic kidney disease, hypertension, kidney transplantation, living kidney donor, long-term follow-up, risk factors

## Abstract

Introduction

Kidney transplantation remains the treatment of choice for end-stage kidney disease (ESKD) because of its excellent outcomes in terms of both graft and recipient survival. However, preserving the long-term health of living kidney donors represents a major challenge in nephrology. Rigorous post-donation follow-up is essential for the early detection of potential renal and cardiovascular complications and for ensuring donor safety.

Methods

We conducted a retrospective, descriptive, and analytical study over a 26-year period (1998-2024) at the Ibn Sina University Hospital Center in Rabat, Morocco. The study included all living-related kidney donors (LRKD) who underwent nephrectomy for kidney donation according to the Kidney Disease: Improving Global Outcomes (KDIGO) eligibility criteria.

The primary objective was to evaluate the incidence of chronic kidney disease (CKD) after donation. Secondary objectives included the assessment of cardiovascular complications, particularly hypertension, and the identification of associated risk factors. Estimated glomerular filtration rate (eGFR) was used to monitor renal function over time.

Quantitative variables were expressed as mean±standard deviation, while qualitative variables were presented as frequencies and percentages. Univariate and multivariate analyses were performed, with statistical significance set at p<0.05.

Results

A total of 154 LRKD were included, with a mean age of 43±12 years and a predominance of female donors (male-to-female ratio: 0.45).

During follow-up, infectious complications were the most frequently observed adverse events, affecting 38 donors (24.6%), with recurrent urinary tract infections being the predominant manifestation, reported in 17 donors (11%). In addition, hypertension was diagnosed in 17 donors (11%), while CKD developed in eight donors (5.2%)

A decline in eGFR below 60 mL/min/1.73 m² was observed in eight donors (5.2%), with no progression to ESKD. The mean serum creatinine at 20 years of follow-up was 82.3±18.56 µmol/L.

Multivariate analysis identified advanced age, male sex, and baseline eGFR as significant predictors of post-donation CKD (p<0.001), whereas no factor was significantly associated with the occurrence of hypertension.

In addition, six full-term pregnancies were reported among female donors, without maternal or fetal complications.

Conclusion

Our findings highlight the importance of rigorous and long-term medical follow-up in LRKD in order to anticipate late complications. Particular attention should be paid to male donors, who appear to be at higher risk of developing renal impairment. Post-donation follow-up should be individualized to ensure optimal management and preserve donor health over time.

## Introduction

Kidney transplantation remains the treatment of choice for end-stage kidney disease (ESKD), as it significantly improves both survival and quality of life compared with dialysis [1]. Living donor kidney transplantation provides better outcomes than deceased donor transplantation, particularly among elderly recipients, as demonstrated by several European studies and meta-analyses [2,3]. These findings have contributed to the growing expansion of living kidney donation worldwide.

However, the proportion of living donor transplantation varies considerably across regions. Approximately 39% of kidney transplants worldwide are performed using living donors, whereas this proportion exceeds 90% in some developing countries due to sociocultural factors and the shortage of deceased donor grafts [4]. At the same time, the increasing number of living donors has been accompanied by an older and more comorbid donor profile, while concerns regarding long-term donor safety continue to limit the development of living donation programs in several countries [5].

Available evidence suggests that the long-term morbidity associated with kidney donation remains relatively low. Carefully selected living donors have an overall survival comparable to that of the general population [6-8], and the risk of ESKD generally remains below 1% at 15 years despite a relative increase in risk compared with healthy non-donors [5,9]. The most frequently reported complications are hypertension and microalbuminuria, whose incidence appears moderate and is influenced by factors such as age, body mass index (BMI), and baseline renal function [5,10].

The 2017 Kidney Disease: Improving Global Outcomes (KDIGO) guidelines emphasize the importance of rigorous donor evaluation, individualized risk assessment, and long-term post-donation follow-up in order to optimize donor safety [11].

In this context, this study aimed to evaluate, over a 26-year period, the incidence of chronic kidney disease (CKD) and cardiovascular complications among living-related kidney donors (LRKD) and to identify the associated risk factors.

## Materials and methods

Study design and population

We conducted a retrospective, descriptive, and analytical study in the Department of Nephrology, Dialysis and Kidney Transplantation at the Ibn Sina University Hospital Center in Rabat, Morocco. The study included 154 LRKD who underwent nephrectomy for kidney donation over a 26-year period between 1998 and 2024.

Data collection

Clinical and demographic data were retrospectively collected from medical records, transplantation registries, and the department's electronic databases.

Inclusion and Exclusion Criteria

All LRKD who underwent donor nephrectomy at the Ibn Sina University Hospital Center between January 1998 and December 2024 were included in the study. No donors were excluded from the analysis, as complete baseline and follow-up data were available through the transplantation registry and archived medical records.

The variables analyzed included demographic characteristics (age and sex), medical history (hypertension, diabetes mellitus, cardiovascular disease, and dyslipidemia), clinical and biological parameters (blood pressure, serum creatinine, estimated glomerular filtration rate (eGFR), proteinuria, fasting blood glucose, and BMI), perioperative data (including surgical and hemorrhagic complications), as well as follow-up outcomes such as the occurrence of hypertension, CKD, recurrent urinary tract infections, pneumonia, survival status, and specific events including post-donation pregnancies.

Post-donation follow-up was conducted through regular outpatient consultations, allowing longitudinal clinical and biological assessment of donors over time.

Donor selection process

All donor candidates underwent a standardized pre-donation evaluation in accordance with national recommendations. This assessment included a detailed medical history, a comprehensive physical examination, and an evaluation of cardiovascular and metabolic risk factors.

Renal function was assessed through repeated measurements of serum creatinine, combined with eGFR using the Modified Diet in Renal Disease (MDRD) equation. Proteinuria was initially screened using urine dipstick testing and subsequently quantified when present.

All candidates underwent renal imaging, including ultrasonography and computed tomography angiography, as well as a complete biological assessment. Cardiovascular evaluation was based on clinical examination, electrocardiography, and additional investigations when indicated.

Infectious disease screening and psychosocial evaluation were also systematically performed. Candidates with uncontrolled hypertension, complicated diabetes mellitus, significant proteinuria, or impaired renal function were excluded from donation.

Definitions of variables

The following are the variables used and their respective definitions: CKD is defined according to the KDIGO criteria as a persistent eGFR <60 mL/min/1.73 m² for at least three months after kidney donation. Hypertension is defined as systolic blood pressure ≥140 mmHg and/or diastolic blood pressure ≥90 mmHg on repeated measurements during follow-up or the use of antihypertensive medication. Proteinuria is defined as urinary protein excretion >300 mg/24 h or a positive urine dipstick test confirmed on follow-up evaluation. Recurrent urinary tract infections are defined as the occurrence of at least two documented episodes. Perioperative or postoperative complications included hemorrhagic, infectious, and respiratory complications.

Statistical analysis

Quantitative variables were expressed as mean±standard deviation, whereas qualitative variables were presented as frequencies and percentages. A univariate analysis was performed to identify factors associated with the studied outcomes. Comparisons were carried out using the chi-squared test for qualitative variables and Student's t-test for quantitative variables. Variables with a p-value of <0.20 in univariate analysis were included in a multivariate logistic regression model to identify independent factors associated with the occurrence of CKD and hypertension. Results were expressed as odds ratios (ORs) with their corresponding 95% confidence intervals (95% CI). A p-value of <0.05 was considered statistically significant. Overall survival was analyzed using Kaplan-Meier survival curves. Statistical analysis was performed using the jamovi software.

## Results

Our study included 154 LRKD. The mean age at the time of donation was 43±12 years, with a predominance of female donors (male-to-female ratio: 0.45). Most donors were siblings, 68 (44.2%), or parents, 53 (34.4%), of the recipients, followed by spouses, 23 (14.9%), whereas other familial relationships were less frequently represented, 10 (6.5%). At the time of donation, the mean serum creatinine was 66.2±11.1 µmol/L, with a mean eGFR of 103±18.1 mL/min/1.73 m². The mean BMI was 26±3.7 kg/m² (Table 1).

**Table 1:** Baseline demographic, clinical, and biological characteristics of the 154 living-related kidney donors at the time of donation Data are presented as mean±SD or N (%).

Variables	Value
Demographic data	
Age (years), mean±SD	43±12
Sex ratio: male/female	0.45
Relationship to recipient, N (%)	
Siblings	68 (44.2%)
Parents	53 (34.4%)
Spouse	23 (14.9%)
Others	10 (6.5%)
Biological parameters, mean±SD	
Creatinine (µmol/L)	66.2±11.1
Estimated glomerular filtration rate (mL/min/1.73 m²)	103±18.1
Body mass index (kg/m²)	26±3.7

Perioperative complications were rare and were observed in four patients (2.6%), including one case of hemorrhagic shock, one colonic injury, one inferior vena cava injury, and one intraoperative hematoma.

**Figure 1:**
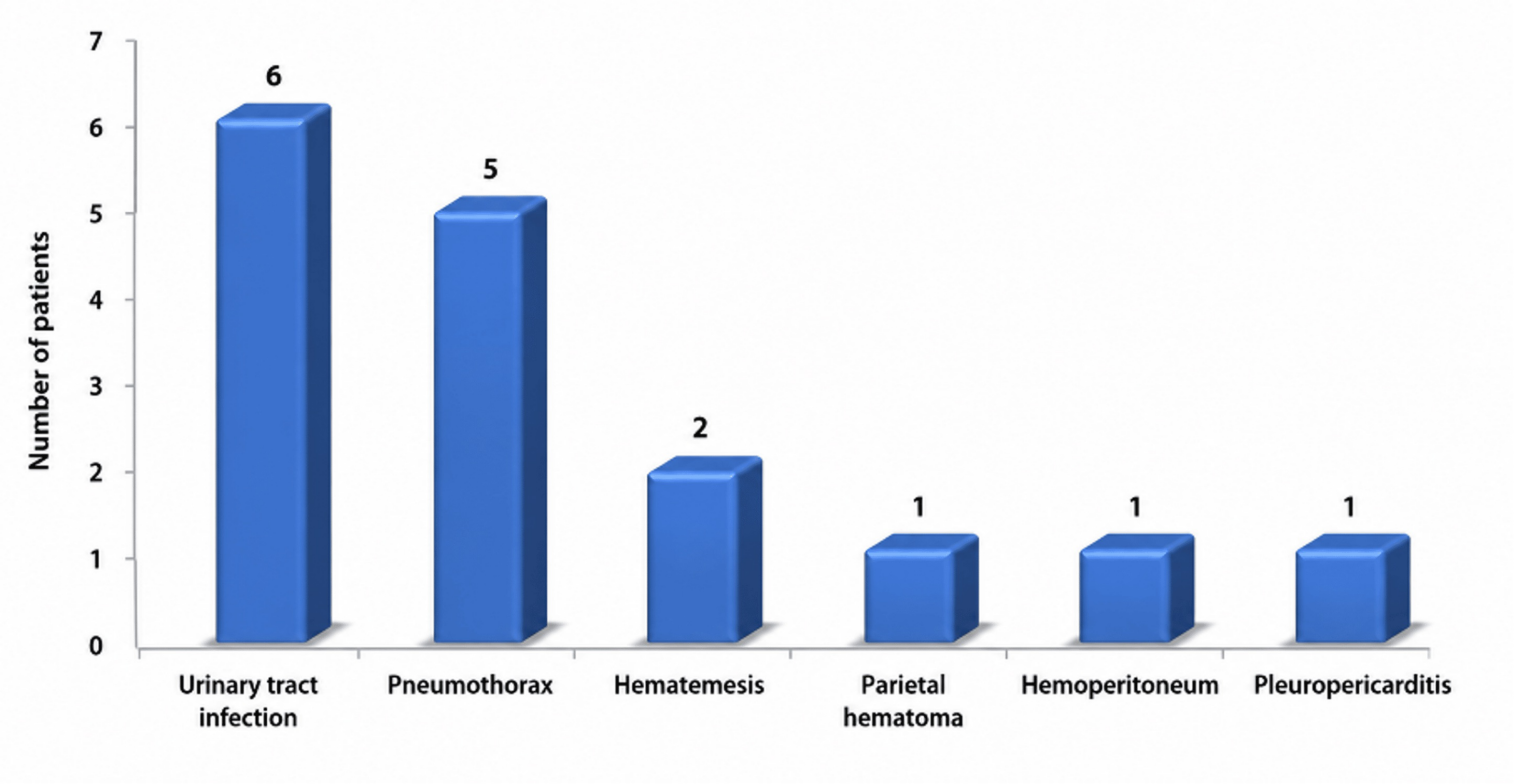
Immediate postoperative complications observed among the 154 living-related kidney donors following donor nephrectomy

The immediate postoperative course was marked by the occurrence of complications in several patients. Hemorrhagic complications were reported in four patients (2.6%), including one parietal hematoma, one hemoperitoneum, and two cases of hematemesis secondary to intubation. Pneumothorax occurred in five patients (3.2%), while one patient (0.6%) developed pleuropericarditis. In addition, six patients (3.9%) experienced postoperative urinary tract infections (Figure 1).

No thromboembolic or urological complications, particularly urinary leakage or hematuria, were observed in our series.

After a mean follow-up of 6.1 years, hypertension developed in 17 patients (11%), including 14 women and three men, with a mean age of 53±9 years. The mean time to onset of hypertension was 9±5 years. The mean systolic and diastolic blood pressure values were 160 mmHg and 100 mmHg, respectively. Thirteen patients were managed with monotherapy, whereas four required dual antihypertensive therapy based on calcium channel blockers, renin-angiotensin-aldosterone system inhibitors, or angiotensin-converting enzyme inhibitors, used alone or in combination.

CKD was identified in eight patients (5.2%), with a median time to onset of 12 months. The mean eGFR was 52.9±5.14 mL/min/1.73 m². No patient presented with significant proteinuria.

Long-term complications were predominantly infectious, occurring in 38 patients (24.6%). Urinary tract infections were the most frequent infectious complication, affecting 17 patients (44.7%), with 1-3 episodes per patient. These infections were mainly caused by Gram-negative bacilli, with* Escherichia coli *being the most commonly isolated pathogen. Cutaneous and mucosal infections were reported in 13 patients (34.2%), predominantly related to* Staphylococcus aureus*.

Surgical complications were observed in 10 patients (6.5%) and included seven incisional hernias, two bone fractures, and one case requiring parathyroidectomy.

**Figure 2:**
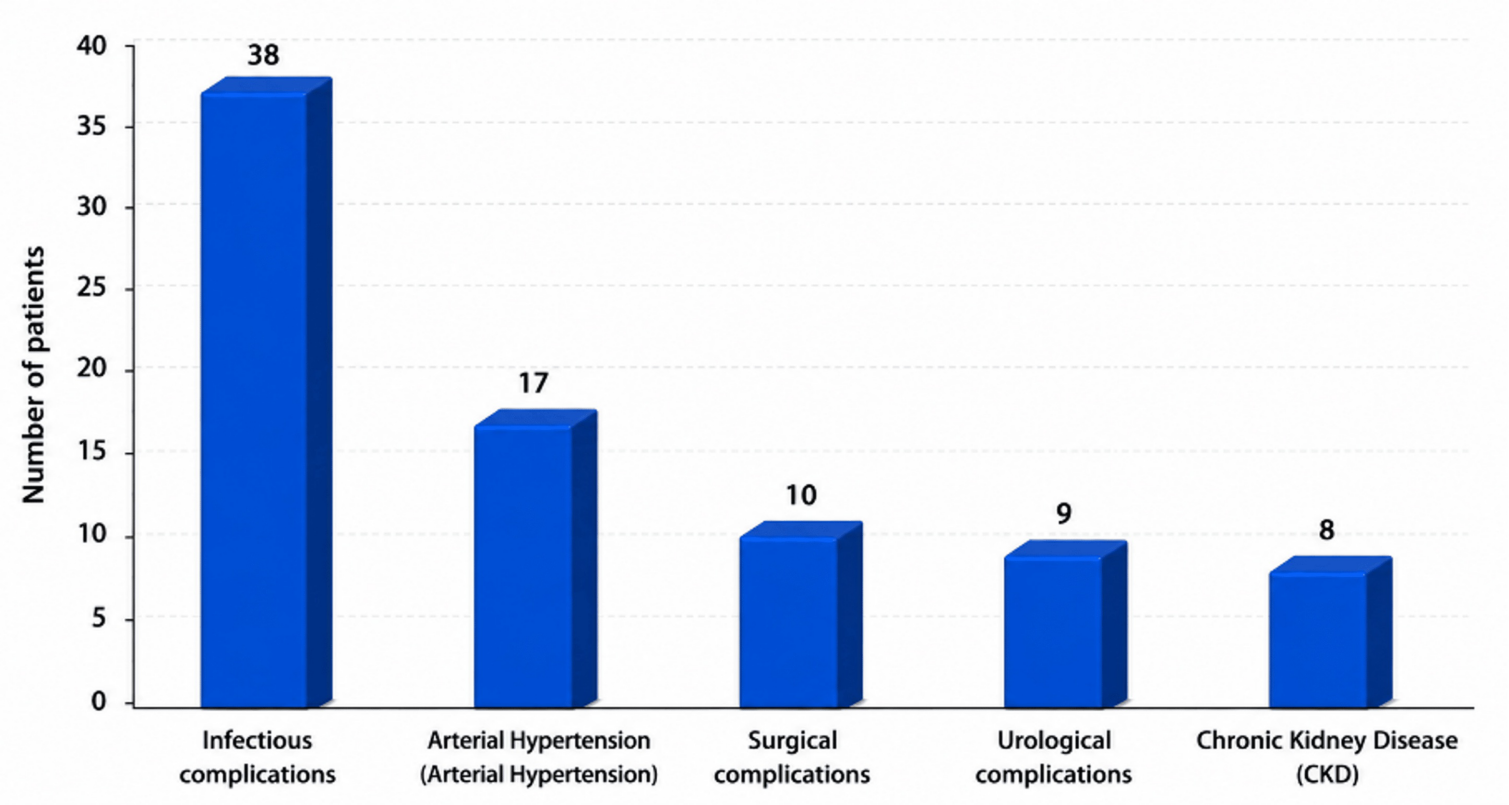
Long-term complications observed during the follow-up of 154 living-related kidney donors

Urological complications occurred in nine patients (5.8%), predominantly benign prostatic hyperplasia (n=6). One case of prostatic adenocarcinoma, one case of calyceal lithiasis involving the remaining kidney, and one case requiring tumorectomy of the native kidney were also reported (Figure 2).

In addition, two cases of malignancy were identified, including one breast cancer and one cervical carcinoma.

**Figure 3:**
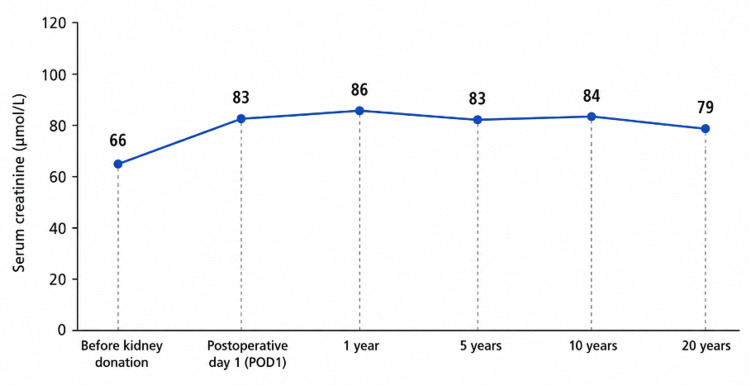
Longitudinal evolution of the mean serum creatinine levels during 20 years of follow-up after living kidney donation

Serum creatinine levels remained stable over the long term, with a mean value of 79±16 µmol/L throughout the 20 years following kidney donation (Figure 3).

Univariate analysis identified several factors significantly associated with the occurrence of post-donation CKD, including older age at donation (OR=1.10; p=0.009), male sex (OR=0.135; p=0.017), smoking status (OR=8.16; p=0.009), and the occurrence of infectious complications (OR=0.931; p=0.008). In contrast, arterial hypertension, baseline glomerular filtration rate, and hemorrhagic complications were not significantly associated with CKD occurrence.

After adjustment in multivariate analysis, only advanced age (OR=1.08; p=0.056), male sex (OR=0.03; p=0.01), and lower baseline glomerular filtration rate (OR=0.88; p=0.021) remained independently associated with post-donation CKD. Smoking and infectious complications lost statistical significance after adjustment, suggesting the presence of confounding factors (Table 2).

**Table 2:** Univariate and multivariate logistic regression analyses identifying the predictors of post-donation CKD among living-related kidney donors Univariate and multivariate logistic regression analyses of factors associated with post-donation CKD among living kidney donors. OR and 95% CI are reported. CKD was defined as an eGFR <60 mL/min/1.73 m² persisting for at least three months. eGFR: estimated glomerular filtration rate; OR: odds ratio; CI: confidence interval; CKD: chronic kidney disease

Variables	Univariate analysis			Multivariate analysis		
	OR	95% CI	P-value	OR	95% CI	P-value
Age at transplantation	1.10	1.02-1.18	0.009	1.08	0.998-1.19	0.056
Female vs. male sex	0.135	0.03-0.69	0.017	0.03	0.002-0.44	0.01
Arterial hypertension	4.08	0.05-18.36	0.992	3.35	0.001-671.35	0.996
Smoking	8.16	1.70-39.25	0.009	0.82	0.08-9.21	0.879
Baseline glomerular filtration rate	0.673	0.15-2.94	0.599	0.88	0.79-0.99	0.021
Infectious complications	0.931	0.88-1.02	0.008	0.36	0.06-2.39	0.295
Hemorrhagic complications	4.17	0.02-47.46	0.994	3.08	0.00009-10938	0.997

Regarding arterial hypertension, none of the studied variables, including age, sex, dyslipidemia, smoking status, BMI, and CKD, showed a statistically significant association in either univariate or multivariate analysis (Table 3).

**Table 3:** Univariate and multivariate logistic regression analyses identifying the predictors of post-donation arterial hypertension among living-related kidney donors Univariate and multivariate logistic regression analyses identifying the predictors of post-donation arterial hypertension among living-related kidney donors. OR with 95% CI are presented. Hypertension was defined as a systolic blood pressure ≥140 mmHg, a diastolic blood pressure ≥90 mmHg, and/or the use of antihypertensive medication. CKD was defined as a persistent eGFR <60 mL/min/1.73 m². BMI: body mass index; OR: odds ratio; CI: confidence interval; CKD: chronic kidney disease; eGFR: estimated glomerular filtration rate

Variables	Univariate analysis			Multivariate analysis		
	OR	95% CI	P-value	OR	95% CI	P-value
Age at transplantation	1.018	0.976-1.063	0.399	1.004	0.957-1.054	0.297
Female vs. male sex	2.28	0.624-8.331	0.212	2.03	0.476-8.715	0.338
Dyslipidemia	0.118	0.007-1.974	0.137	7.94	0.343-182.39	0.196
Smoking	1.59	0.029-35.17	0.993	5.67	0.036-27.41	0.992
BMI	0.988	0.863-1.130	0.863	0.977	0.836-1.145	0.781
CKD	2.91	0.538-15.97	0.214	6.914	0.856-56.00	0.07

**Figure 4:**
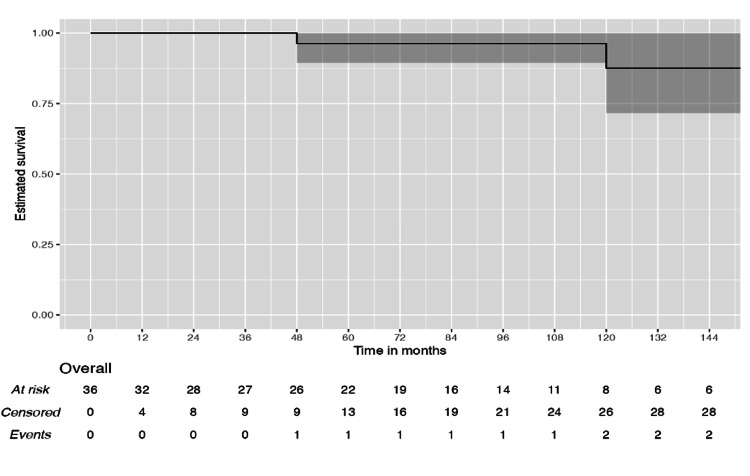
Kaplan-Meier overall survival curve of living-related kidney donors during follow-up

The estimated overall five-year survival rate was 96.3% (Figure 4).

**Figure 5:**
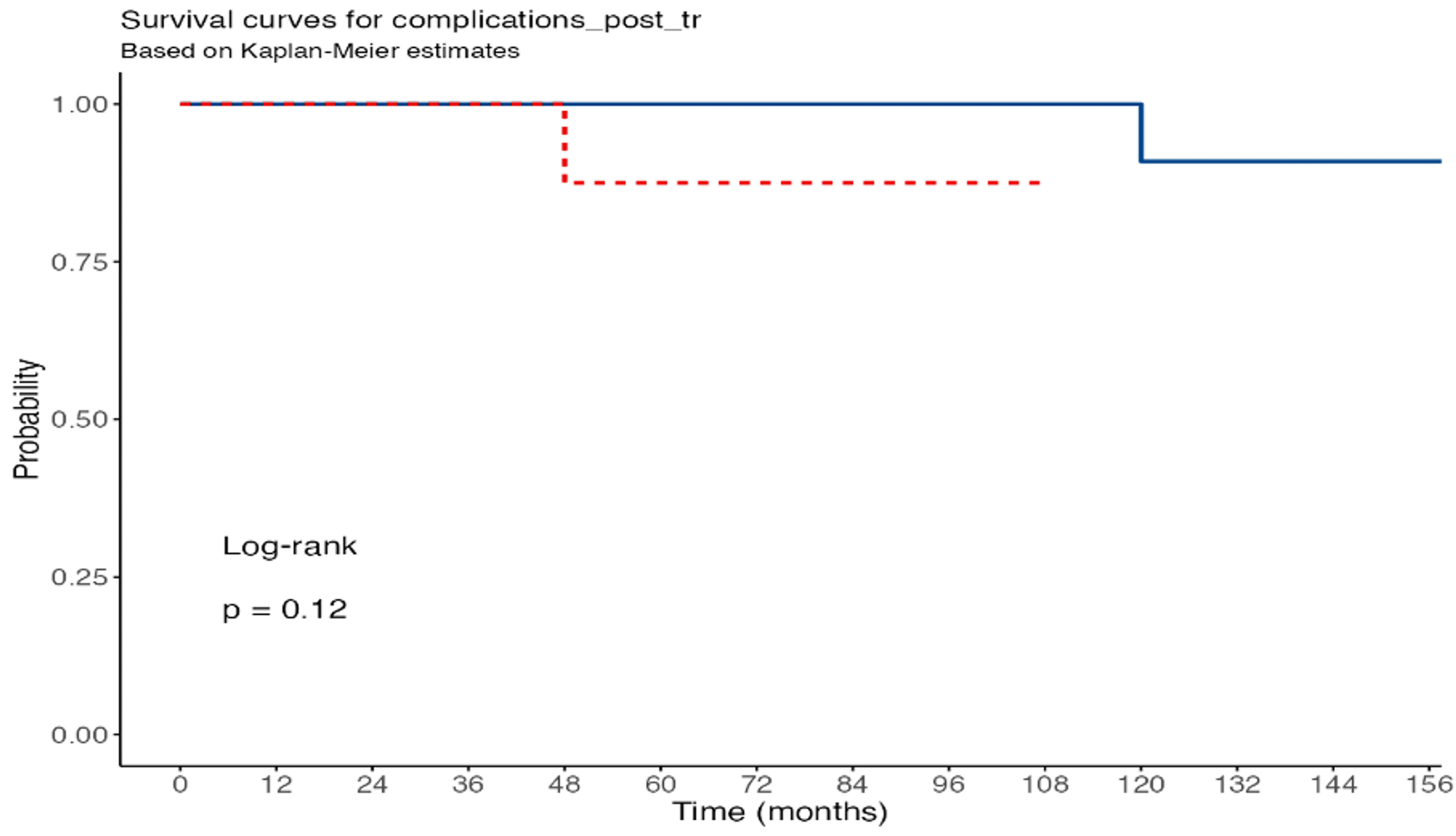
Kaplan-Meier survival curves according to the occurrence of post-donation complications

**Figure 6:**
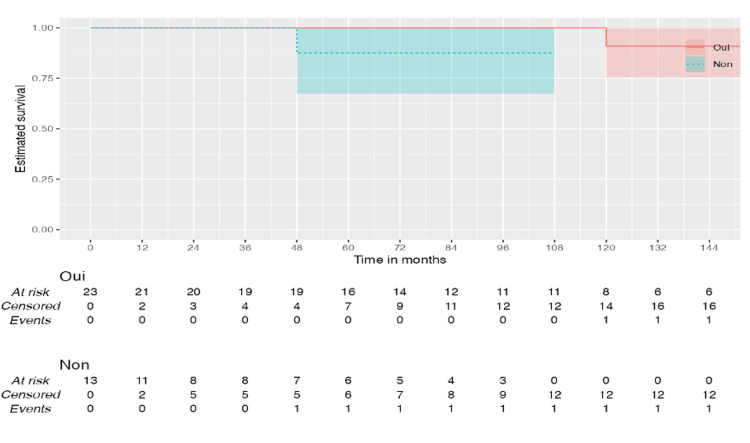
Kaplan-Meier survival curves according to the occurrence of complications

Comparison of survival curves between patients who developed complications and those who did not revealed no statistically significant difference in mortality (p=0.12) (Figures 5-6).

## Discussion

In our cohort of 154 LRKD, the mean age at donation was 43±12 years, with a predominance of female donors (male-to-female ratio: 0.45). After a mean follow-up of 6.1 years, de novo hypertension developed in 17 donors (11%), while CKD occurred in eight donors (5.2%). No donor progressed to ESKD. Infectious complications were observed in 38 donors (24.6%) and urological complications in nine donors (5.8%), representing the most common long-term adverse events. Perioperative complications were uncommon, occurring in four donors (2.6%).

Multivariate analysis identified older age, male sex, and lower baseline eGFR as independent predictors of CKD, whereas no variable was significantly associated with the occurrence of hypertension.

Our findings suggest a low renal and cardiovascular morbidity associated with living kidney donation. The incidence of hypertension in our cohort (11%) was lower than that reported by Ibrahim et al. (32%) after a mean follow-up of 12 years [7] and by Fernandes et al., who observed hypertension in 30% of Portuguese donors, with rates reaching 21% at five years and more than 50% at 15 years of follow-up [5]. This difference may be explained by the shorter follow-up duration in our study (six years) and by differences in demographic characteristics, as our cohort was younger.

Similarly, in the Turkish study by Güneş et al., 20.2% of donors developed hypertension, and 18.9% exhibited an eGFR <60 mL/min/1.73 m², although none progressed to ESKD [4]. Our results, showing CKD in 5.2% of donors without progression to ESKD, are overall consistent with these findings and further support the concept that nephron mass reduction is generally well tolerated in carefully selected donors.

Predicting the risk of post-donation ESKD and CKD remains challenging. The risk projection model developed by Grams et al. estimated that the average 15-year probability of ESKD among carefully selected donors is approximately 0.3%, with variations according to age, sex, and ethnicity [8]. The review by Fleetwood et al. further reported that, for most donors, the risk of kidney failure remains below 1% at 15 years and that living donors have only slightly higher risks than healthy non-donors while still presenting lower risks than those observed in the general population [3]. Similarly, a recent mini-review published in Frontiers highlighted that fewer than one in 200 donors develop ESKD and that the absolute risk is estimated at approximately 30 per 10,000 donors at 15 years [3].

However, a Norwegian cohort study including 1,901 donors demonstrated an 11.4-fold higher risk of ESKD among donors compared with healthy individuals eligible for donation but who did not donate. Nevertheless, the absolute incidence remained very low, and the excess risk was mainly observed among biologically related donors sharing common genetic susceptibility with recipients [12].

Our findings confirm that lower baseline eGFR, advanced age, and male sex are associated with the occurrence of CKD after donation, in agreement with the observations of Ibrahim et al., who reported that age and BMI were associated with both reduced eGFR and the development of hypertension [7]. The predictive value of baseline eGFR was also emphasized in the Turkish study, where lower pre-donation eGFR independently predicted a post-donation eGFR <60 mL/min/1.73 m² [4].

Post-donation hypertension was not associated with demographic or biological factors in our cohort, in contrast to the findings of Fernandes et al., who identified elevated BMI, dyslipidemia, and higher pre-donation blood pressure as independent predictors of hypertension [5]. The pathophysiological mechanisms underlying hypertension after nephrectomy remain a matter of debate. Proposed mechanisms include glomerular hyperfiltration, activation of the renin-angiotensin-aldosterone system, and alterations in vascular tone.

Experimental studies suggest that nephron mass reduction induces compensatory increases in glomerular pressure and renal plasma flow, which may contribute over time to the development of hypertension and albuminuria. However, distinguishing the direct effects of nephrectomy from those related to aging and conventional cardiovascular risk factors, such as weight gain, sedentary lifestyle, and smoking, remains challenging [13].

Our results highlight a relatively high rate of long-term infectious and urological complications. Recurrent urinary tract infections represented the most frequent complication (44.7% of all infectious events), consistent with other cohorts in which infections accounted for the majority of post-donation complications [4].

The occurrence of malignancies, including breast cancer, cervical carcinoma, and prostatic adenocarcinoma, remained uncommon and cannot be directly attributed to donor nephrectomy.

Finally, six full-term pregnancies were reported in our cohort without maternal or fetal complications, which is reassuring. Nevertheless, recent studies have described a modest increase in the risk of hypertensive disorders during pregnancy among living kidney donors, highlighting the importance of close obstetrical follow-up in this population [3].

Despite its retrospective single-center design, our study provides valuable long-term data on living kidney donor outcomes over a 26-year period in a North African population, a region for which published data remain limited. Large cohort studies have demonstrated that perioperative mortality is extremely low and that donor survival is comparable to that of matched healthy controls [6]. Furthermore, the cumulative risk of ESKD remains below 0.5% [14], while the excess risk reported in certain studies appears to be largely related to hereditary factors [12].

In our series, two donors died from causes unrelated to kidney donation, and no case of ESKD was observed, further supporting the concept that living kidney donation is a safe and encouraging practice.

These findings strongly support living kidney donation by demonstrating that donor generosity contributes to saving lives while preserving donors' own long-term health. They provide an important argument for reassuring potential donors and promoting living kidney donation, an altruistic act whose benefits largely outweigh its risks, as demonstrated by numerous international studies [12,14].

Nevertheless, despite these reassuring outcomes, several studies have highlighted that concerns regarding long-term risks, insufficient information, and sociocultural barriers may limit the development of living donation programs [12,14,15]. This underlines the importance of providing clear information to potential donors, ensuring appropriate counseling, and implementing structured long-term follow-up programs [16,17].

The development of educational programs and rigorous long-term monitoring strategies, in accordance with KDIGO recommendations, together with the establishment of national donor registries, may further improve donor safety and support the expansion of living kidney donation [11,13].

## Conclusions

Living kidney donation was associated with favorable long-term outcomes, with low rates of CKD, hypertension, complications, and mortality. Advanced age, male sex, and lower baseline eGFR were identified as predictors of post-donation renal impairment. These findings support the safety of living kidney donation and highlight the importance of careful donor selection and long-term follow-up.
